# The Intraplantar CFA Inflammatory Pain Model Incompletely Captures Pain Comorbidities Across Sexes

**DOI:** 10.1002/jnr.70157

**Published:** 2026-09-19

**Authors:** Caitlin E. Fenech, Neda Assareh, Karin R. Aubrey

**Affiliations:** ^1^ Pain Management Research Laboratories Kolling Institute Royal North Shore Hospital, Northern Sydney Local Health District St Leonards New South Wales Australia; ^2^ School of Medical Sciences, Faculty of Medicine and Health University of Sydney Sydney New South Wales Australia; ^3^ Sydney Pain Consortium, Faculty of Medicine and Health University of Sydney Sydney New South Wales Australia; ^4^ Brain and Mind Centre, Faculty of Science University of Sydney Sydney New South Wales Australia; ^5^ Kolling Institute, Faculty of Medicine and Health University of Sydney Sydney New South Wales Australia

**Keywords:** animal behavior, anxiety, complete Freund's adjuvant, nociception, preclinical pain model, sex difference

## Abstract

In humans, chronic pain is characterized by a combination of heightened nociceptive sensitivity along with emotional comorbidities, with more women impacted than men. A reported limitation of preclinical models is that they often fail to encapsulate this complexity, and few studies have directly compared behavioral outcomes between male and female mice. The intraplantar Complete Freund's adjuvant (CFA) inflammatory pain model is a widely used preclinical model of persistent mechanical and thermal hypersensitivity, which triggers sex‐dependent adaptations in neuronal signaling. However, the literature regarding the duration of pain hypersensitivity and the presence or absence of associated locomotor and anxiety‐like changes is inconsistent. Therefore, this study aimed to systematically assess the CFA model's capacity to duplicate pain comorbidities in male and female mice. CFA‐injected or saline control male and female mice were assessed over 3 weeks for mechanical and cold nociceptive sensitivity (von Frey and acetone test), locomotion (open field test), and anxiety‐like behaviors (light–dark and elevated plus maze tests). CFA‐injected mice of both sexes reliably developed mechanical hypersensitivity and cold allodynia. Transient locomotor deficits were observed only in male mice at early timepoints, and no changes in anxiety‐like behaviors in either males or females were observed. Hence, the CFA persistent pain model is a practical tool for inducing pronociceptive states in male and female mice but does not reliably produce anxiety‐like comorbidities over the tested time periods, suggesting that additional factors may be necessary to elicit these behavioral outcomes.

## Significance Statement

Preclinical models provide mechanistic insights to guide safer, more effective treatments in the clinic. The intraplantar CFA‐model is a widely used model of persistent inflammatory pain, supporting drug validation and identifying biological and central nervous system processes that contribute to chronic pain states. However, protocol variability and the predominant use of male animals affect its reproducibility and confound interpretation. Additionally, reports of anxiety‐like effects are inconsistent. This study directly compares nociceptive and anxiety‐like behaviors in male and female mice under standardized conditions, providing robust data for pain researchers and highlighting both limitations and opportunities for refining use of the CFA model.

## Graphical abstract

This study provides a standardized investigation into nociceptive and anxiety‐like behaviors in the intraplantar Complete Freund's Adjuvant (CFA) inflammatory pain model in both male and female mice across a 3‐week period. We found robust changes in mechanical hypersensitivity and cold allodynia but did not see any changes in anxiety‐like behaviors.

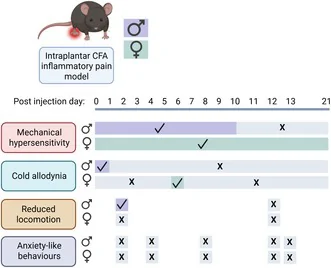

## 1 Introduction

Chronic pain is a highly prevalent and debilitating health condition, affecting approximately one in five people (Treede et al. 2015). Despite its widespread impact, effective treatment options remain limited; current pharmacotherapies provide only partial relief in about half of patients (Dworkin et al. 2010). Compounding this issue, many individuals with chronic pain develop comorbid conditions such as depression, anxiety, and sleep disturbances. These challenges highlight the need for a deeper understanding of how nociception and affective (emotional) behaviors, and their circuits, interact. Preclinical pain research using animal models of persistent pain strives to provide this fundamental knowledge, to aid in the development of safer and more effective new chronic pain treatments. However, the evidence produced by pain models is often inconsistent, particularly regarding the emergence of chronic pain comorbidities, thereby limiting data interpretation, clinical relevance, and translational potential (Soliman and Denk 2024).

Complete Freund's adjuvant (CFA) injection into the hind paw of rodents is a widely used preclinical model for studying persistent inflammatory pain. It reliably induces mechanical and heat hypersensitivity in the injected paw, which is responsive to analgesics (Knight et al. 1992; Stein et al. 1988). These nociceptive effects typically persist for 3–4 weeks (Burek et al. 2022; Wei et al. 2025), although reported durations vary: some studies observe hypersensitivity lasting as little as 2 weeks, while others report effects extending up to 6 weeks (Baumbach et al. 2024; Liu et al. 2015; Pitzer et al. 2016, 2019; Sheahan et al. 2017; Burek et al. 2021; Knight et al. 1992; Urban et al. 2011).

Beyond nociceptive changes, several studies have documented CFA‐induced deficits in general locomotion and anxiety‐like behaviors over a time course that parallels the nociceptive effects (Burek et al. 2021; Cardenas et al. 2021; Chen et al. 2013; Hofmann et al. 2017; Liu et al. 2015; Narita et al. 2006; Parent et al. 2012; Pitzer et al. 2016, 2019; Refsgaard et al. 2016; Sheahan et al. 2017; Zhou et al. 2019). These findings suggest the model may be useful for exploring the broader neurological and behavioral comorbidities associated with prolonged pain. However, other studies have failed to detect significant locomotor or anxiety‐like alterations, highlighting inconsistencies in the model's ability to replicate these complex outcomes (Flores‐García et al. 2025; Gaspar et al. 2021; Urban et al. 2011).

Additionally, to date, the majority of preclinical pain studies have used male mice, with systematic inclusion of females only gaining traction in recent years (Burek et al. 2022; Kilkenny et al. 2010; Wei et al. 2025). As a result, there is a notable scarcity of data comparing CFA‐injected and control groups in female animals for both nociceptive and affective behaviors (Cardenas et al. 2021; Flores‐García et al. 2025; Papadogiannis and Dimitrov 2022; Pitzer et al. 2019; Refsgaard et al. 2016; Zhang et al. 2024). Indeed, only one study has simultaneously assessed nociception and anxiety‐like behaviors in female C57BL/6 mice, the most used mouse strain in pain research (Pitzer et al. 2019). This lack of data is especially striking given the well‐documented sex differences in immune responses and neuronal signaling following CFA administration (Bouchet et al. 2023; McPherson et al. 2023; Sorge et al. 2015; Tonsfeldt et al. 2016). Despite this, it remains unclear whether these immunological differences translate into measurable behavioral outcomes. Addressing this knowledge gap is critical, especially considering that inflammatory chronic pain conditions, such as rheumatoid arthritis, affect more women than men (Greenspan et al. 2007; Mogil 2012).

Some of the variability and conflicting results reported by different research groups are likely related to known confounds. First, the injury model (i.e., volume injected into the hind paw, diluted versus undiluted CFA), experimental design, handling, and apparatus habituation schedules are not consistently carried out or reported across the studies (Burek et al. 2021; Segelcke et al. 2023; Sensini et al. 2020; Sorge et al. 2014). In addition, a recent systematic review and meta‐analysis uncovered significant variability in behavioral outcomes across animal species, strain and sourcing (Burek et al. 2022), and other studies have revealed there is a substantial influence of housing and testing conditions on behavioral outcomes, both between and within cages (Pitzer et al. 2016; Zhang et al. 2024; Khanna et al. 2019).

This study aims to concurrently assess the nociceptive, locomotion, and anxiety‐like behaviors, behavioral outcomes of hind paw CFA injection in male and female mice, keeping strain, source, species, housing conditions, researcher, and handling protocols consistent. Building a systematic repository of replicable data across multiple relevant behavioral outcomes in commonly used chronic and persistent pain models is necessary to highlight the strengths and limitations of each model. Such efforts will improve interpretation of model‐derived data and generate testable hypotheses, enhance relevance and translatability, and ultimately support more effective preclinical‐to‐clinical translation in pain research.

## 2 Methods

### 2.1 Animals

All experiments were conducted in accordance with the Australian Code for the Care and Use of Animals for Scientific Purposes and approved by the University of Sydney Animal Ethics Committee (Protocol: 2022/2085). Adult male (*n* = 16) and female (*n* = 16) mice (8–9 weeks, C57Bl/6J), obtained from the institutional colony (Kearns Animal Facility), were housed in ventilated cages (up to four/cage), maintained on a 12/12 h light/dark cycle (lights on at 0700 and off at 1900), 23°C, 70% humidity with food (Gordons Rat & Mouse Premium 23% Protein Diet) and water provided *ad libitum*. Cages were enriched with a house igloo, tissues for nesting, and paddle pop sticks. Mice were regularly monitored for general health and well‐being and weighed twice a week. All experiments reported in this paper were carried out by the same female researcher.

### 2.2 Study Design and Randomization

**FIGURE 1:**
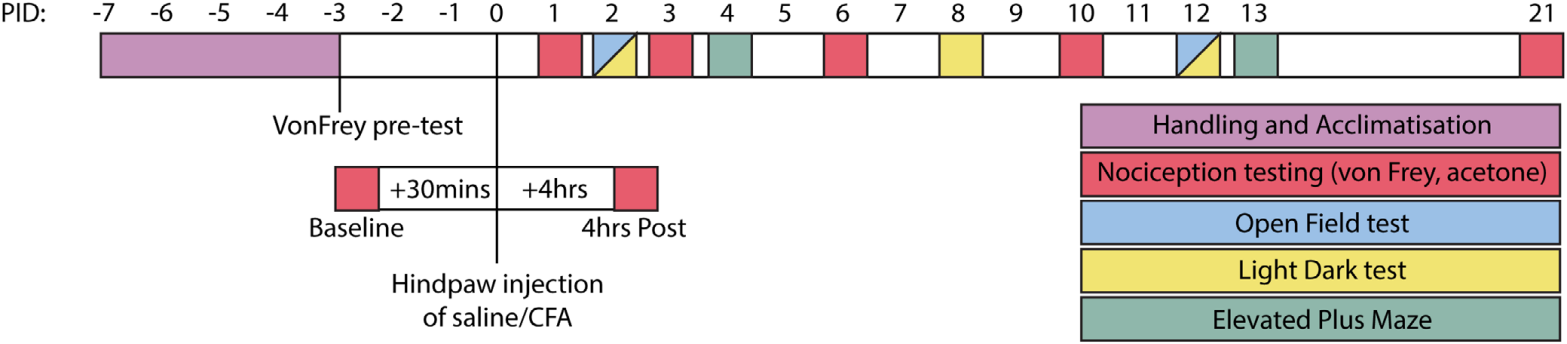
Experimental timeline. After a 5‐day handling and acclimatization period, male and female mice underwent baseline nociception testing (von Frey and acetone test), before a hind paw injection of saline or CFA. Over the next 3 weeks, animals underwent nociception testing 4 h post injection and on PID 1, 3, 6, 10, and 21, the open field test on PID 2 and 12, the light–dark test on PID 2, 8 and 12, and the elevated plus maze test on PID 4 and 13. CFA, complete Freund's adjuvant; PID, post injection day.

An extensive 3‐week behavioral assay was designed to compare early and late‐stage behavioral effects of the CFA‐injection (Figure 1) and assess the effect of intraplantar CFA injection over time. Group sizes were determined a priori using estimates of variability and treatment effects reported in a previous study employing the intraplantar CFA model in male and female mice of the same strain (Pitzer et al. 2019). To maintain adequate statistical power in the event of animal loss, exclusion or larger variability, the calculated sample size of *n* = 6 animals per group was increased by 25%. Animals were excluded from analysis if the von Frey pre‐test thresholds were lower than 0.2 g (Sheahan et al. 2017), however no animals met this exclusion criteria. Animals were randomly allocated to treatment groups (i.e., saline or CFA) in cage groups (all cage mates were of the same treatment group; *n* = 8/group). All nociception behavioral testing occurred around midday, except for baseline and 4 h post CFA timepoints. The mouse testing order was alternated to avoid the confound of time of day or circadian rhythm effects across testing sessions. CFA administration results in visually obvious paw edema, which prevents a fully blinded experimental design, however analysis was completed with the researcher blinded to treatment group allocation. The nociceptive testing data for PID 15 were excluded from analysis because only four data points were collected due to experimenter illness.

### 2.3 Handling and Habituation

All mice were handled by the experimenter for a period of 5 days preceding any behavioral testing. The handling consisted of gently lifting the mouse by the base of the tail onto the experimenter's wrist and transferring the mouse between their hands for 1 min. The last 3 days of the handling protocol were paired with 10 min of acclimatization to the experimental room and 1 min habituation to the von Frey/acetone stand (dim red light 610–630 nm, < 3 Lux; (Khanna et al. 2019)). On the 5th day, the mice underwent a von Frey pre‐test to reduce test‐related stress. By the conclusion of the 5‐day handling protocol, none of the mice exhibited an obvious stress response (no shaking, vocalization, or jumping) to handling by the experimenter, the room, or the apparatus.

### 2.4 CFA‐Induced Inflammatory Pain State

The CFA‐model of persistent inflammatory pain was used in this study (Knight et al. 1992; Stein et al. 1988). Mice were anesthetized with 2%–3% isoflurane (1 L/min O_2_) and sedation was confirmed by the absence of a toe pinch reflex. Mice then received a single unilateral intraplantar left hind paw injection of 20 μL undiluted CFA (F5881‐Sigma‐Aldrich). Similarly, control mice were injected unilaterally in the left hind paw with 20 μL of 0.9% sterile saline.

### 2.5 Nociceptive Tests

On the testing day, animals were placed in elevated Perspex enclosures with wire mesh bases and acclimatized to the testing environment for 30 min (dim red light 610–630 nm at < 3 Lux; (Khanna et al. 2019)). Two nociceptive tests were conducted in these enclosures: von Frey (mechanical hypersensitivity) and acetone (cold allodynia), with a 5‐min interval between tests. (Anderson et al. 2014; François et al. 2017; Mitchell et al. 2021).

*von Frey* (*mechanical hypersensitivity*): Mechanical allodynia was assessed by applying a series of mechanical forces with graded von Frey filaments for mice (2.83, 3.22, 3.61, 3.84, 4.08, 4.17, 4.31, 4.56, and 4.74 corresponding to mechanical forces between 0.2 and 6.84 g; North Coast Medical, San Jose, CA, USA) pressed perpendicularly against the injected (left) hind paw and held for approximately 2 s. Testing began with the 4.08 (1.2 g) von Frey hair then proceeded to a higher or lower force depending upon the lack or presence of a withdrawal response, respectively, for a total of five times. Withdrawal threshold was determined using the simplified up‐down method (SUDO), calculated from the value of the fifth presentation of a von Frey hair with an adjustment factor of ±0.5, such that:

$$\mathrm{PWT}=F_{5}\pm \left[0.5\times \left(F_{5}-F_{\mathrm{adj}}\right)\right]$$

where $F_{5}$ is the force (*g*) of the filament applied on the fifth presentation, $F_{\mathrm{adj}}$ is the force (*g*) of the adjacent filament immediately below $F_{5}$ (for a response) or immediately above $F_{5}$ (for a non‐response). If the animal responds to the fifth filament, the subtraction term was used while the addition term was used when there was no response (Bonin et al. 2014). For example, if the animal responded on the fifth presentation to the 1.2 g force filament (4.08), and the force of the next lighter filament (3.84) is 0.69 g, then the calculated paw withdrawal threshold (*g*) is 1.2 − (0.5 × (1.2–0.69)) = 0.95 g. To fit the data for parametric analysis, the paw withdrawal threshold values were then log‐transformed (Mills et al. 2012).

*Acetone* (*cold allodynia*): To test cold responses, 20 μL of acetone was sprayed on the center of the injected (left) hind paw and the number of licks was recorded over a 30 s period (Yoon et al. 1994). If the mouse licked its paw for an extended period, one lick per 2 s was noted (i.e., 4 s of continuous licking was counted as two licks). The test was repeated once on the same paw with at least 5 min between tests. The final value used for statistical analysis was the average of the number of licks between the two tests.

### 2.6 Locomotion and Anxiety Testing

*Open field* (*locomotor activity and anxiety‐like behaviors*): Locomotor activity was measured using the open field test. After a 10‐min acclimatization period to the testing room, the mice were individually placed in an enclosed open‐top arena (50 cm × 50 cm × 50 cm, dim white light (30 ± 10 Lux)). An overhead camera recorded their behavior for a 15‐min period. This period is sufficient to monitor both locomotion and anxiety‐like behaviors (Bailey and Crawley 2009). Between tests, 20% ethanol was used to clean the apparatus to remove odor cues. Rearing and jumping responses were manually scored. Measurement of the total distance traveled (m), average speed (m/s), time in the center zone (s), number of entries into the center zone, and time mobile (s) was obtained with ANY‐Maze video tracking software (version 7; Stoelting Co.). Centre zone was classified as the middle 25 cm × 25 cm zone.

*Light–Dark* (*anxiety‐like behaviors*): The light–dark box test (LD box, dark chamber: 20 cm wide × 18 cm × 30 cm high; light chamber (60 ± 10 lux): 28 cm wide × 27 cm long × 30 cm high) was used to test anxiety‐like behaviors (Bourin and Hascoët 2003). After a 10‐min acclimatization period to the testing room (dim white light, 30 ± 10 Lux), the mice were individually placed in the light chamber of the LD box facing the entry to the dark chamber (~18 cm from entrance) and allowed to explore freely for 5 min. An overhead camera recorded their behavior, and the following measures were obtained following ANY‐Maze video tracking software analysis (version 7; Stoelting Co.): number of entries into the light zone, time in the light zone (s), and total distance traveled in the light zone (m). Between animals, 20% ethanol was used to clean the apparatus to remove odor cues.

*Elevated plus maze* (*anxiety‐like behaviors*): The elevated plus maze (EPM; two 60 cm arms, 5 cm wide with 15 cm high walls) was used to support the findings in the LD test (Riebe and Wotjak 2012; Walf and Frye 2007). After a 10‐min acclimatization period to the testing room under dim white light (40 ± 10 lux), the animals were individually placed in the center of the elevated plus maze facing a closed arm. An overhead camera recorded their behavior for a 5‐min period. The time spent (s) in the open and closed arms was measured using ANY‐Maze video tracking software (version 7; Stoelting Co.). Between animals, 20% ethanol was used to clean the apparatus to remove odor cues.

Tests were conducted in a sequence designed to reduce potential stress effects (Figure 1; Pitzer et al. 2019).

### 2.7 Statistical Analysis

All data was analyzed using GraphPad Prism (version 10, GraphPad Software Inc., La Jolla, CA) and presented as mean ± SEM. Significance, as indicated by asterisks, was defined as *p* < 0.05 a priori. For all datasets, a Shapiro–Wilk test and visual inspection of a *Q*–*Q* plot were performed to test for normality. Significance was analyzed via a repeated measures two‐way ANOVA, followed by Bonferroni's multiple comparison test if the main effect of CFA injection was significant or there was a significant interaction between time and CFA injection. A detailed statistical summary can be found in Table S1. Behavioral test schematics were created with BioRender.

## 3 Results

### 3.1 CFA‐Induced Persistent Inflammatory Pain Causes Transient Nociceptive Hypersensitivity

**FIGURE 2:**
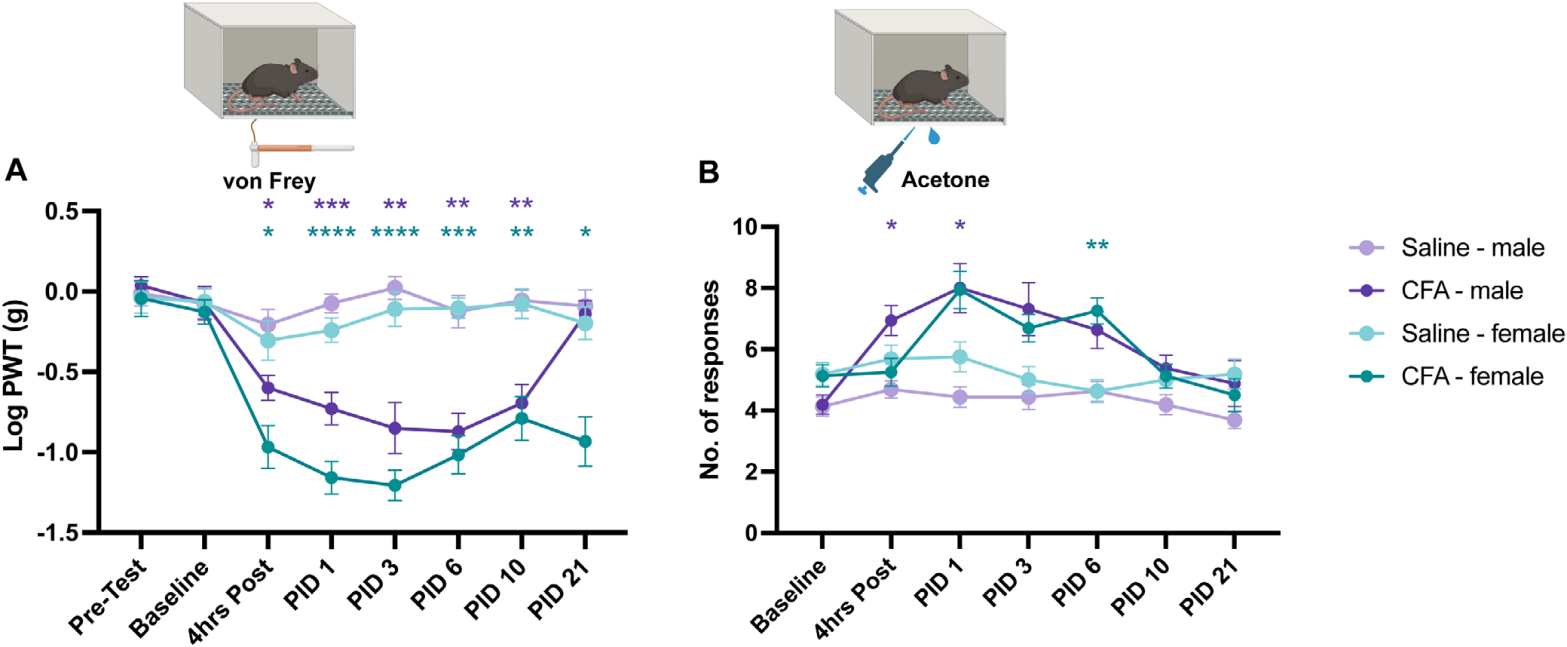
CFA‐induced inflammatory pain induces mechanical sensitivity and cold allodynia in male and female mice. CFA‐injection significantly reduced paw withdrawal thresholds in the von Frey test for both males and females up to 10 days post injection when compared to saline‐injected mice. This difference was maintained at PID 21 in females (A). In the acetone test, CFA‐injected male mice showed more responses than saline‐injected male mice 4 h post‐CFA, and on PID 1, while this difference was seen only on PID 6 for females (B). CFA, complete Freund's adjuvant; PID, post‐injection day; PWT, paw withdrawal threshold. Values are presented as mean ± SEM (*n* = 8 for all groups) and significance was analyzed via a repeated‐measures two‐way ANOVA with a post hoc Bonferroni correction; *p < 0.05; **p < 0.01; ***p < 0.001; ****p < 0.0001.

To test the effect of persistent inflammatory pain induced by hind paw injection of CFA, nociceptive tests were conducted (von Frey, acetone) in male and female mice and compared to saline injection. The tests were conducted 4 h post injection and repeated on post injection day (PID) 1, 3, 6, 10, and 21 to measure the progression of the pain state across a 3‐week period. Mechanical sensitivity was measured using the von Frey test. A von Frey pre‐test was conducted prior to baseline testing to habituate the animals to the test, and no significant difference was found between the pre‐test and baseline values for any of the groups. CFA‐injected male and female mice exhibited significantly lower withdrawal thresholds than saline‐injected mice 4 h post‐CFA injection as well as on PID 1, 3, 6, and 10. Three weeks after CFA injection (PID 21), withdrawal thresholds resolved back to baseline in male mice but remained lower in female mice (Figure 2A). Next, the acetone test was carried out to measure CFA effects on cold allodynia. CFA‐injected male mice showed significantly more responses than saline‐injected mice 4 h post‐CFA injection and on PID 1, while this difference was seen only at PID 6 for female CFA‐injected mice (Figure 2B).

Overall, CFA induced robust mechanical hypersensitivity from 4 h post‐CFA injection until PID 10 in animals of both sexes. However, hypersensitivity remained evident beyond PID 21 in female mice. In contrast, cold allodynia was detected only at selected timepoints between 4 h post‐CFA injection and PID 6 in CFA‐injected mice of both sexes and was not detectable at PID 21.

CFA injection has been reported to have inconsistent effects on locomotor and anxiety‐like behaviors across a wide range of studies (Burek et al. 2021, 2022; Cardenas et al. 2021; Chen et al. 2013; Flores‐García et al. 2025; Gaspar et al. 2021; Hofmann et al. 2017; Liu et al. 2015; Narita et al. 2006; Parent et al. 2012; Pitzer et al. 2016, 2019; Refsgaard et al. 2016; Sheahan et al. 2017; Urban et al. 2011; Wei et al. 2025; Zhou et al. 2019). Further, there is a dearth of evidence in female mice. Thus, we compared the behavior of saline‐ and CFA‐injected male and female mice using the open field test, light–dark test, and elevated plus maze during the 2 weeks following CFA injection, when mechanical hypersensitivity and cold allodynia are present.

### 3.2 Male, but Not Female, CFA‐Injected Animals Have a Transient Reduction in Locomotion in the Open Field Test

**FIGURE 3:**
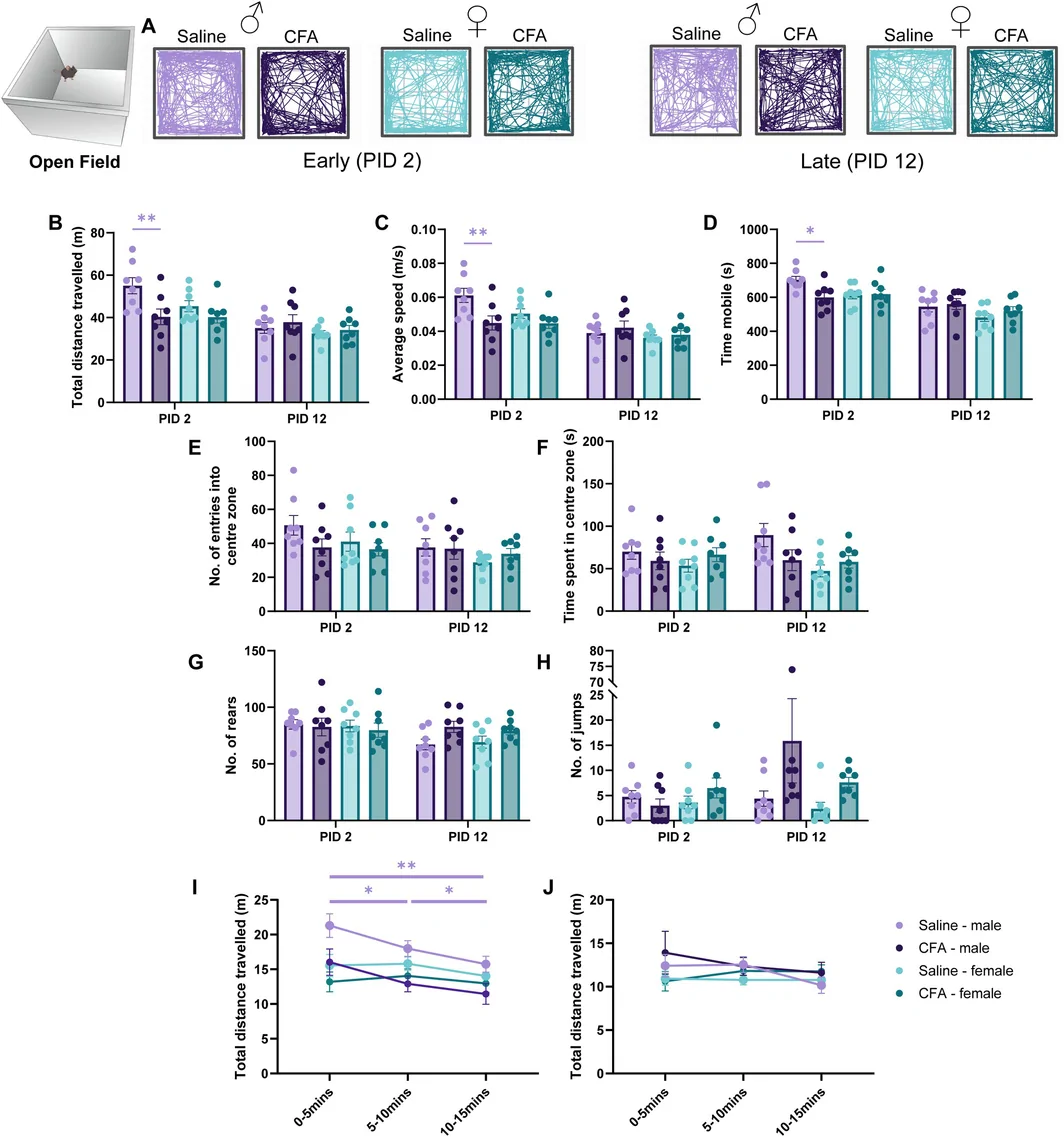
CFA‐induced inflammatory pain transiently reduces open field locomotor activity in male mice at PID 2. Track plots of center point in the open field of saline‐ (light purple/light green) and CFA‐injected (dark purple/dark green) male (purple) and female (green) mice at PID 2 and PID 12 (A). Locomotor behavior (B–D), was significantly reduced in male CFA‐injected mice at PID 2 only, while center zone activity (E, F) and rearing (G) and jumping (H) behaviors were unchanged. Total distance traveled (m) in the 15‐min open field was split into 5‐min time intervals and saline‐injected male mice showed habituation to the arena at PID 2 (I) while no habituation effect was seen at PID 12 for any of the groups (J). CFA: Complete Freund's adjuvant, PID, post‐ injection day. Data for *n* = 8 animals/group are indicated on the graphs and values are presented as mean ± SEM. Significance was analyzed via a repeated‐measures two‐way ANOVA with a post hoc Bonferroni correction, *p < 0.05; **p < 0.01.

Locomotor behavior (distance traveled, average speed, mobility, center zone entries/duration, rearing, and jumping behavior) over a 15‐min open field test was quantified at PID 2 and PID 12 (Figure 3A). On PID 2, CFA‐injected male mice exhibited reduced locomotor activity relative to saline‐injected controls, indicated by changes in total distance (Figure 3B), average speed (Figure 3C), and mobility (Figure 3D). However, no significant locomotor behavioral differences were detected in females or for center zone activity, rearing, and jumping behaviors at any of the timepoints (Figure 3E–H). To see if CFA intraplantar injection affected exploratory behaviors and habituation to the open field environment (Bailey and Crawley 2009), locomotor activity over the 15‐min open field test was analyzed in 5‐min intervals. At PID 2, we found a significant reduction of distance traveled in the open field exclusively in the saline‐injected male mice over the time course of the open field (Figure 3I,J).

Overall, CFA injection resulted in a sex‐dependent reduction in locomotor activity in exclusively male mice at PID 2.

### 3.3 CFA‐Induced Persistent Pain Is Not Associated With Changes in Anxiety‐Like Behavior in Male and Female Mice

To directly assess anxiety‐like behaviors, multiple related behavioral assays are recommended to enhance the reliability of findings and minimize the influence of confounding factors associated with individual tests (Riebe and Wotjak 2012). Therefore, we carried out both the light–dark (LD) test and the elevated plus maze (EPM) in male and female CFA‐ and saline‐treated mice.

**FIGURE 4:**
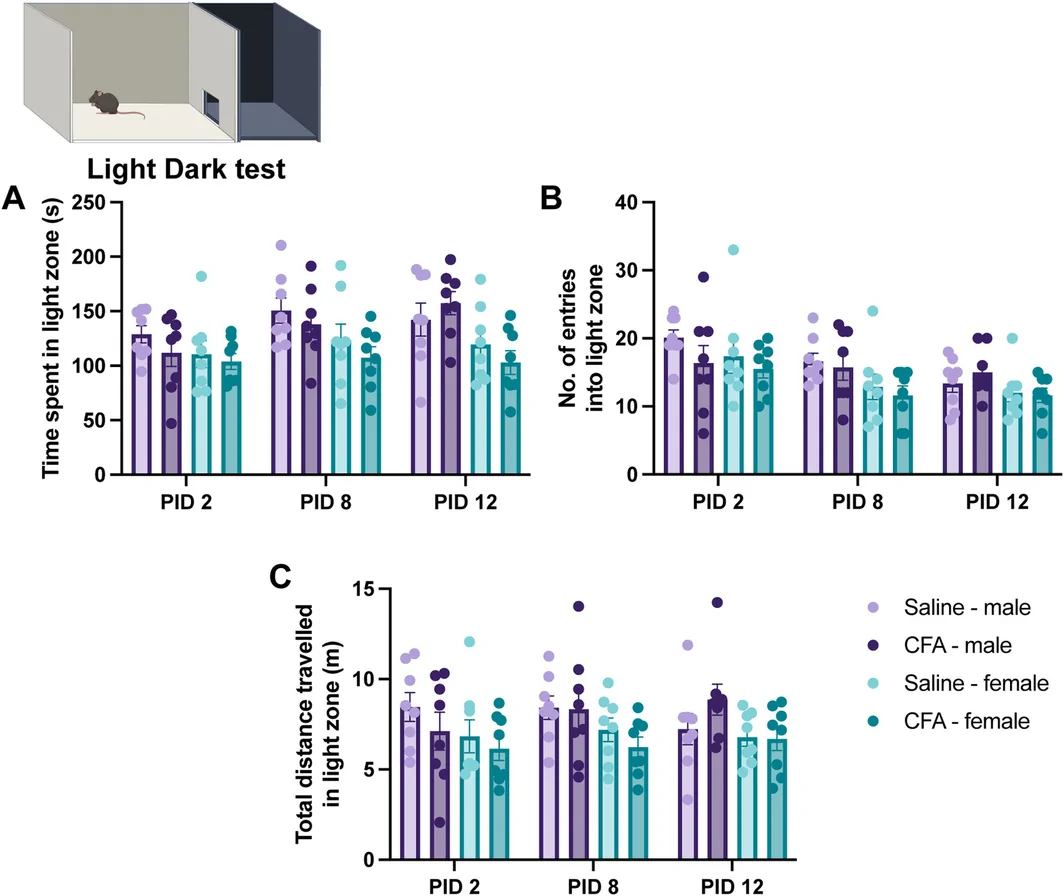
CFA‐induced inflammatory pain is not associated with any changes in light dark test behaviors in male or female mice. Light–dark behaviors were compared in saline and CFA‐injected male and female mice at PID 2, PID 8 and PID 12. Behavior was assessed over 5 min and time in light zone (A, D), number of entries (B) and distance traveled (C) was quantified. CFA, complete Freund's adjuvant, PID, post‐injection day. Data for *n* = 8 animals/group are indicated on the graphs and values are presented as mean ± SEM. Significance was analyzed via a repeated‐measures two‐way ANOVA with a post hoc Bonferroni correction.

For LD, time spent or number of entries into the light zone and distance traveled between the CFA‐ and saline‐injected mice of either sex at PID 2, PID 8, and PID 12 was analyzed. There was no significant effect of CFA for any of the behaviors measured (Figure 4A–C). Total distance travelled in the light zone was analyzed to ensure PID 2 locomotor activity changes due to CFA did not interfere with the light zone measurements. Overall, there was no main effect of CFA intraplantar injection observed in the light‐dark test at any timepoints.

**FIGURE 5:**
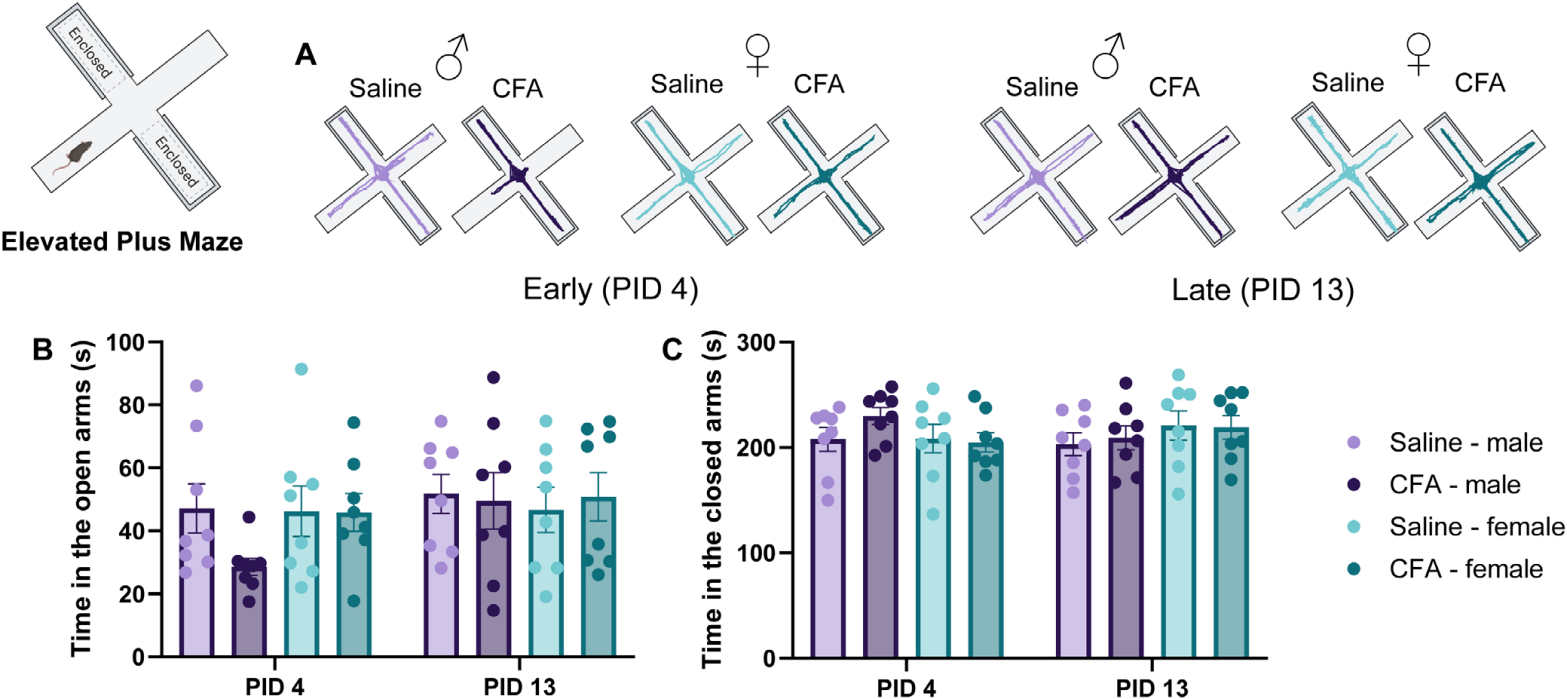
CFA‐induced inflammatory pain is not associated with any changes in time spent in open or closed arms in the elevated plus maze in male or female mice. Elevated plus maze track plots of saline and CFA‐injected male and female animals at PID 4 and PID 13 (A). Behavior was assessed over 5 min and time in open (B) and closed arms (C) quantified. CFA, complete Freund's adjuvant; PID; post‐injection day. Data for *n* = 8 animals/group are indicated on the graphs and values are presented as mean ± SEM. Significance was analyzed via a repeated‐measures two‐way ANOVA with a post hoc Bonferroni correction.

In the EPM test (Figure 5A), time spent in the open and closed arms at PID 4 and PID 13 was analyzed. Consistent with the LD data, there was no significant main effect of CFA for time spent in either the open arms or closed arms at either timepoint (Figure 5B,C). Total distance travelled was analyzed over the EPM testing period and no changes in locomotor activity were detected (not shown).

## 4 Discussion

### 4.1 Summary and Methodological Considerations

This study used a systematic approach to compare nociceptive and affective behaviors in the CFA model in both male and female mice. We found that mechanical sensitivity is reliably increased following CFA hind paw injection compared to saline injection, with male mice recovering to baseline levels and females remaining hypersensitive at 3 weeks post‐CFA. For cold allodynia responses, increases in nocifensive responses to acetone were measured in males at 4‐h and 1‐day post CFA, and in females at PID 6, after which time it resolved. The open field test revealed that CFA‐injected males have reduced locomotor activity at early timepoints. In tests to assess CFA‐induced changes in anxiety‐like behaviors, no behavioral changes were detected in either male or female mice in the LD or EPM tests over the testing period. Thus, we conclude the CFA‐induced inflammatory pain model reliably induces transient nociceptive hypersensitivity in both males and females and transient locomotor deficits in males only. Further, we found no evidence that anxiety‐like changes can be detected over the 2 weeks following CFA‐injection in either males or females (Table 1).

**TABLE 1:** Summary of behavioral responses altered following CFA.

Behavior	CFA effect	
	Males	Females
Mechanical hypersensitivity	↑ (4 h post‐PID 10)	↑ (4 h post‐PID 21)
Cold allodynia	↑ (4 h post‐PID 1)	↑ (PID 6)
Locomotion	↓ (PID 2)	X
Anxiety‐like behaviors	X	X

Abbreviation: PID, post injection day.

The major strength of the CFA‐induced persistent inflammatory pain model is its accessibility and reproducibility in generating robust nociceptive hypersensitivity, mainly measured through the von Frey and Hargreaves test (Burek et al. 2021; Cardenas et al. 2021; Chen et al. 2013; Cobos et al. 2012; Flores‐García et al. 2025; Gaspar et al. 2021; Kandasamy et al. 2016; Liu et al. 2015; Narita et al. 2006; Papadogiannis and Dimitrov 2022; Parent et al. 2012; Pitzer et al. 2016, 2019; Refsgaard et al. 2016; Rivera‐García et al. 2024; Sheahan et al. 2017; Urban et al. 2011; Y. Zhang et al. 2024; Zhou et al. 2019). Importantly, in contrast to more complex models such as neuropathic pain models (Bennett and Xie 1988; Seltzer et al. 1990), it requires minimal technical expertise (i.e., no surgical procedures are involved) and induces peak hypersensitivity within a short timeframe. In addition, CFA‐induced nociceptive changes cause measurable alterations in inflammatory state (Sorge et al. 2015), and spinal and descending pain‐pathway signaling (Hryciw et al. 2021; Tonsfeldt et al. 2016; Yu et al. 2021). As a result, the CFA‐inflammatory pain model offers practical advantages for preclinical research, limiting animal distress, engaging canonical peripheral, spinal and central pathways, and streamlining experimental timelines.

Undiluted CFA was used in the present study to induce persistent inflammatory pain, consistent with several previous reports (Refsgaard et al. 2016; Sheahan et al. 2017; Urban et al. 2011; Ghasemlou et al. 2015). However, other studies have used diluted CFA preparations. Although inconsistent findings regarding anxiety‐like behaviors have also been reported across studies regardless of CFA concentration (Chen et al. 2013; Refsgaard et al. 2016; Narita et al. 2006; Burek et al. 2021; Hofmann et al. 2017), no study has directly compared the effects of diluted and undiluted CFA on both nociceptive and affective outcomes. Nevertheless, available evidence suggests that increasing CFA concentration may exacerbate behavioral and pathological outcomes. For example, higher CFA concentrations have been associated with greater burrowing deficits (Gould et al. 2016) and increased disease severity when used in the adjuvant‐induced arthritis models (Berke et al. 2025). The extent to which CFA concentration influences nociceptive hypersensitivity, locomotor activity, or anxiety‐like behaviors therefore remains unclear. Thus, CFA concentration represents an important experimental variable that may contribute to inconsistencies in the literature and warrants systematic investigation.

### 4.2 Mechanical and Cold Nociceptive Hypersensitivity in CFA‐Treated Male Versus Female Mice

In our study, we report much needed corroborative data for the usefulness of this model in female mice. Further, we provide high‐quality evidence that is not distorted by common methodological variability related to strain, researcher gender, animal housing, handling protocols and experimental timeline (Meseguer Henarejos et al. 2020; Sensini et al. 2020). We confirm that mechanical hypersensitivity develops rapidly and reaches a stable effect size in both male and female mice (Cardenas et al. 2021; Flores‐García et al. 2025; Papadogiannis and Dimitrov 2022; Pitzer et al. 2019; Y. Zhang et al. 2024) with mechanical hypersensitivity resolving more quickly in males compared to females. In contrast, cold allodynia was detectable in both sexes but was characterized by a smaller and less sustained effect. Significant cold hypersensitivity was observed primarily during the early phase following CFA administration and resolved within approximately 1 week, with significance detected only intermittently across time points. These findings are consistent with the notion that different pain modalities are coded through distinct sensory pathways, and that CFA induces alterations in sensory/perceptive pathways that are different across the sexes (Choi et al. 2020; Ding et al. 2025; Hockley et al. 2017; Keay and Bandler 2001; Keay and Bandler 2002; Nguyen et al. 2026; Zhang et al. 1990). The differences in the timeline and duration of measurable nociceptive change should be considered by researchers testing analgesic compounds in this model. Appropriate controls to ensure that testing windows align with the relevant pain modality and animal sex should always be carried out and accurately reported.

### 4.3 Recovery From Nociceptive Hypersensitivity in CFA Treated Male Versus Female Mice

Our data confirms previous findings showing that CFA‐induced nociceptive hypersensitivity typically resolves within 2–3 weeks in male mice, even though paw inflammation persists for considerably longer periods (Knight et al. 1992; Burek et al. 2021). Interestingly, this is not the case in female mice. This mismatch may be explained by known sex differences in immune responses to CFA injection (Sorge et al. 2015). In addition, sex differences in the engagement of compensatory analgesic mechanisms within central pain‐modulatory circuits, such as descending inhibitory pathways, are also likely to play a role (Corder et al. 2013; Severino et al. 2018; Coutens et al. 2026). Recent evidence shows that endocannabinoid (eCB) levels increase in the midbrain periaqueductal gray, a well‐characterized node of the descending pain modulatory pathway, during the 2–3 weeks following CFA treatment, and that inhibition of eCB signaling reinstates nociceptive hypersensitivity after its resolution (Bouchet et al. 2023; Coutens et al. 2026). The timing of this increase is temporally associated with the recovery from mechanical hypersensitivity and cold allodynia observed here. Together, these findings suggest that the use of the CFA model offers opportunities to investigate (analgesic) central nervous system adaptations to persistent pain states, as well as the mechanisms underlying the sex‐dependent differences we have observed.

### 4.4 CFA Model and Anxiety‐Like Behaviors

Our finding that neither male nor female CFA‐injected mice displayed alterations in light–dark (LD) or elevated plus maze (EPM) behavior during the period of peak nociceptive hypersensitivity is consistent with the only other study to directly compare open field, EPM, and LD behaviors in male and female CFA‐injected C57BL/6 mice (Pitzer et al. 2019). We specifically focused on the first 2 weeks following CFA injection because this represents the timeframe when nociceptive changes are present and thus has been widely used in studies employing the CFA model. In addition, it is also a period in which reports of anxiety‐like behaviors are varied and inconsistent—with some studies reporting anxiety‐like behaviors during the weeks following CFA injection, while others report no effect (Burek et al. 2021; Cardenas et al. 2021; Chen et al. 2013; Flores‐García et al. 2025; Gaspar et al. 2021; Hofmann et al. 2017; Liu et al. 2015; Narita et al. 2006; Parent et al. 2012; Pitzer et al. 2016, 2019; Refsgaard et al. 2016; Sheahan et al. 2017; Urban et al. 2011; Zhou et al. 2019).

More broadly, a temporal separation between sensory and affective outcomes is a common feature across rodent models of persistent and chronic pain. Nociceptive hypersensitivity and allodynia typically develop rapidly, often within hours to days, whereas anxiety‐like behaviors generally emerge after weeks or months of ongoing pain (Burgess et al. 2002; Suzuki et al. 2007; Yalcin et al. 2011; Llorca‐Torralba et al. 2019; Hestehave et al. 2024). Interestingly, some studies have reported that anxiety‐like behaviors are measurable when testing is conducted several weeks or months after CFA injection, long after nociceptive hypersensitivity has resolved (Narita et al. 2006; Parent et al. 2012; Zhou et al. 2019). While the finding of anxiety‐like phenotypes at these later timepoints remains variable (Burek et al. 2021), these observations suggest that CFA‐injection continues to influence central nervous system function long after nociceptive hypersensitivity has peaked or resolved. This again highlights the importance of considering testing timeframe when interpreting behavioral outcomes in this model.

Given the high prevalence of anxiety and depression among individuals with chronic pain, this temporal lag between nociceptive and affective outcomes has often been viewed as a limitation of the CFA model and other preclinical chronic pain models (Mogil 2009). However, a similar mismatch occurs in humans, where emotional comorbidities become more common after prolonged periods of pain rather than immediately following injury (Yalcin et al. 2014; Aaron et al. 2025). The delayed onset of anxiety‐like behaviors therefore may not necessarily represent a failure of animal models to capture the human condition but rather reflect a conserved biological process that is present across species.

One unifying explanation is that persistent pain states in isolation may not be sufficient to directly induce robust anxiety‐like behavior, but instead serve to “sensitize” or “prime” stress‐ and fear‐related neural circuits. Consistent with this interpretation, chronic pain models are associated with changes indicative of negative affect and stress, including reduced nesting, increased grimace scores, behavioral avoidance, and altered physiological stress responses (Seo et al. 2006; Kwon et al. 2008; Negus et al. 2015; Refsgaard et al. 2016; Mogil 2020). Our open field exploration/habituation data could be interpreted in this context and suggest that persistent inflammatory pain altered the adaptive response of male mice to a novel environment.

Further evidence for a pain‐induced “priming” stress effect also comes from a recent study using the chronic constriction injury model in mice. Here, nociceptive hypersensitivity was present but anxiety‐like behavior was absent 2 weeks after the CCI injury. However, when animals were exposed to an additional mild to moderate stressor within these 2 weeks, robust anxiety‐like behaviors emerged (Assareh et al. 2025). These findings support the hypothesis that persistent pain may not directly cause anxiety but instead increases susceptibility to future stressors. In this framework, pain acts as a vulnerability factor that amplifies the behavioral consequences of subsequent (or previous) adverse experiences.

### 4.5 Future Opportunities

Extending this concept further, a primed‐network framework may help explain why only a subset of individuals with chronic pain develop clinically significant affective comorbidities. Human studies consistently identify pre‐existing vulnerabilities, including early‐life adversity, prior psychiatric conditions, and ongoing life stressors, as risk factors for poorer pain outcomes and increased rates of anxiety and depression (Aaron et al. 2025; Bussières et al. 2023; Rouch et al. 2024). Similarly, preclinical data show that CFA‐induced behavioral abnormalities are exacerbated in mice exposed to early‐life stress or repeated stressors (Liu et al. 2015). Viewed in this context, the absence of anxiety‐like behavior during the first 2 weeks following CFA injection is not unexpected, and rather than representing a deficiency of the model it may instead reflect a biologically meaningful feature of persistent pain states. Variability in reporting for the presence of anxiety‐like behaviors across CFA‐model studies could therefore arise, at least in part, from differences in prior experience, mild to moderate environmental stressors, or baseline genetic vulnerabilities that interact with pain to determine whether affective disturbances emerge. On this basis, future studies should aim to develop a more nuanced framework for considering pain–affect interactions in the CFA model and comprehensive reporting of housing, handling and husbandry conditions should be systematically reported. Suggested first step experiments should be aimed at determining whether manipulation of descending pain pathways can prevent or prolong the resolution of CFA‐induced hypersensitivity in male and female mice (see Bouchet et al. 2023; Corder et al. 2013; Coutens et al. 2026), and testing whether anxiety‐like behaviors are reliably unmasked in CFA‐injected mice following exposure to mild‐to‐moderate secondary stressors.

## 5 Conclusion

Overall, this study demonstrates that the CFA model of inflammatory pain can be relied upon to produce measurable changes in nociceptive responses in the one to 3 weeks post CFA‐injection but fails to model anxiety‐like behaviors over this time period. Our experiments include both male and female cohorts, in line with recently published guidelines for pain research (Finn et al. 2025), allowing for a much needed analysis of sex‐dependent differences in this model. Notably, we report sex‐dependent changes in recovery from nociceptive hypersensitivity. By employing a methodology that minimizes confounding variables, including consistent handling, habituation schedules, and animal sourcing, we strengthen the reliability and replicability of findings using the intraplantar CFA‐induced inflammatory pain model and in the process highlight its limitations and opportunities for its use in future research.

## Author Contributions

All authors had full access to all the data in the study and take responsibility for the integrity of the data and the accuracy of the data analysis. **C.E.F.**, **N.A.**, **K.R.A.:** conceptualization. **C.E.F.**, **K.R.A.:** writing – review and editing. **C.E.F.**, **N.A.:** methodology. **C.E.F.:** investigation, formal analysis, writing – original Draft, visualization. **K.R.A.:** resources, supervision, funding acquisition.

## Funding

This work was supported by grants from the Pain Foundation Ltd., the Ernest Heine Family Foundation, and an Australian Government Research Training Program (RTP) Scholarship.

## Conflicts of Interest

The authors declare no conflicts of interest.

## Supporting information


**Table S1:** Statistical test details.

**Data S1:** Transparent science questionnaire for authors.

## Data Availability

The data that support the findings of this study are available from the corresponding author upon reasonable request.
